# Graph theoretical analysis of complex networks in the brain

**DOI:** 10.1186/1753-4631-1-3

**Published:** 2007-07-05

**Authors:** Cornelis J Stam, Jaap C Reijneveld

**Affiliations:** 1 Department of Clinical Neurophysiology, VU University Medical Center, De Boelelaan 1117, 1081 HV Amsterdam, The Netherlands; 2 Department of Neurology, VU University Medical Center, De Boelelaan 1117, 1081 HV Amsterdam, The Netherlands

## Abstract

Since the discovery of small-world and scale-free networks the study of complex systems from a network perspective has taken an enormous flight. In recent years many important properties of complex networks have been delineated. In particular, significant progress has been made in understanding the relationship between the structural properties of networks and the nature of dynamics taking place on these networks. For instance, the 'synchronizability' of complex networks of coupled oscillators can be determined by graph spectral analysis. These developments in the theory of complex networks have inspired new applications in the field of neuroscience. Graph analysis has been used in the study of models of neural networks, anatomical connectivity, and functional connectivity based upon fMRI, EEG and MEG. These studies suggest that the human brain can be modelled as a complex network, and may have a small-world structure both at the level of anatomical as well as functional connectivity. This small-world structure is hypothesized to reflect an optimal situation associated with rapid synchronization and information transfer, minimal wiring costs, as well as a balance between local processing and global integration. The topological structure of functional networks is probably restrained by genetic and anatomical factors, but can be modified during tasks. There is also increasing evidence that various types of brain disease such as Alzheimer's disease, schizophrenia, brain tumours and epilepsy may be associated with deviations of the functional network topology from the optimal small-world pattern.

## 1. Background

The human brain is considered to be the most complex object in the universe. Attempts to understand its intricate wiring patterns and the way these give rise to normal and disturbed brain function is one of the most challenging areas in modern science[1]. In particular, the relationship between neurophysiological processes on the one hand, and consciousness and higher brain functions such as attention, perception, memory, language and problem solving on the other hand, remains an enigma to this day. In the last decades of the 20^th ^century significant progress has been made in neuroscience with an essentially reductionistic, molecular biologic research programme [2]. The Nobel prize in physiology or medicine awarded to Eric Kandel in 2000 for discovering the molecular mechanisms of memory in the snale aplysia signifies the importance of this work. However, despite the impressive increase of knowledge in neuroscience in terms of molecular and genetic mechanisms, progress in true understanding has been disappointing, and few theories are available that attempt to explain higher level brain processes.

For this reason there has been increased interest to search for other approaches to study brain processes and their relation to consciousness and higher brain functions [3]. One strategy has been to conceive the brain as a complex dynamical system and to search for new approaches in other fields of science that are also devoted to the study of complex systems. In recent years considerable progress has been made in the study of general complex systems, consisting of large numbers of weakly interacting elements. Three research areas in physics and mathematics have proven to be particularly valuable in the study of complex systems: (i) nonlinear dynamics and related areas such as synergetics; (ii) statistical physics which deals with universal phenomena at phase transitions and scaling behaviour, and (iii) the modern theory of networks, which is derived from graph theory [4].

Nonlinear dynamics has been applied to the study of the brain since 1985, and has become a very active research field in itself [5,6]. Application of nonlinear dynamics to neuroscience has lead to the introduction of new concepts such as attractors, control parameters and bifurcations as well as to the development of a whole range of new analytical tools to extract nonlinear properties from time series of brain activity. This has resulted for instance in new ways to model epileptic seizures as well as methods to detect and perhaps even predict the occurrence of seizures [7-9]. Recently, the focus in studies of nonlinear brain dynamics has shifted from trying to detect chaotic dynamics to studying nonlinear interactions between brain areas [10,11]. The study of critical phenomena and scaling behaviour in brain dynamics has also been very fruitful. Several studies have shown that time series of brain activity demonstrate scaling with characteristic exponents, suggesting critical dynamics near a phase transition [12-15].

The modern theory of networks, which originated with the discovery of small-world networks and scale-free networks at the close of the last millennium is the most recently developed approach to complex systems [16,17]. The study of complex networks has attracted a large amount of attention in the last few years, and has resulted in applications in such various fields as the study of metabolic systems, airport networks and the brain [18-22].

The aim of the present review is to discuss recent applications of network theory to neuroscience. After a brief historical introduction we summarize the basic properties and types of networks, and some important results on the relation between network properties and processes on these networks, in particular synchronization phenomena. Subsequently we will discuss applications to neuroscience under three headings: (i) modelling of neural dynamics on complex networks; (ii) graph theoretical analysis of neuroanatomical networks; (iii) applications of graph analysis to studies of functional connectivity with functional magnetic resonance imaging (fMRI), electroencephalography (EEG) and magnetoencephalography (MEG).

## 2. Historical overview

The modern theory of networks has its roots in mathematics as well as in sociology. In 1736 the famous mathematician Leonard Euler (1707–1783) solved the problem of 'the bridges of Konigsberg'. This problem involved the question whether it is possible to make a walk crossing exactly one time each of the seven bridges connecting the two islands in the river Pregel and its shores. Euler proved that this is not possible by representing the problem as an abstract network: a "graph". This is often considered the first proof in graph theory. Since then graph theory has become an important field within mathematics, and the only available tool to handle network properties theoretically. An important step forward occurred when random graphs were discovered [23,24]. In random graphs connections between the network nodes are present with a likelihood p. Many important theorems have been proven for random graphs. In particular it has been shown that properties of the graphs often undergo a sudden transition ('phase transition') as a function of increasing p. However, despite the success of classical graph theory, it was not a very good or useful theory for real networks encountered in nature. One empirically observed phenomenon that could not be explained by classical theory was the fact the 'distances' in sparsely and mainly locally connected networks were often much smaller than expected.

This phenomenon was probably first observed by the Hungarian writer Frigyes Karinthy in a short story called 'Chains' [25]. In this story he speculates that in the modern world the 'distance' between any two persons is unlikely to be more than five persons. As it turned out, this was a remarkable foresight of an important fact about certain classes of networks. The first person to study this phenomenon more scientifically was Stanley Milgram (1933–1984). He was interested in quantifying distances in social networks. In one experiment he sent letters to randomly chosen subjects in the USA. They were informed that the letter should go to a certain person in Boston. However, the subjects were only allowed to send the letter to another person they knew well, and who might possibly be a little closer to the target in Boston. As it turned out, many letters did reach the target person, and on average each letter was sent only 5.5 times. This experiment could count as the first empirical proof of the 'small-world' phenomenon, also referred to as 'six degrees of separation' [26]. The 'small-world' phenomenon was later confirmed in other experiments (for instance: the letter experiment was repeated with e-mail) but for a long time no satisfactory explanation was available.

This situation changed suddenly in 1998 with the publication of a paper in Nature by Duncan Watts and Steven Strogatz [16]. In this paper the authors proposed a very simple model of a one-dimensional network on a ring. Initially each node ('vertex') in the network is only connected to its k nearest neighbours (k/2 on each side). K is called the degree of the network. Next, with a likelihood p, connections ('edges') are chosen at random and connected to another vertex, also chosen randomly. With increasing p more and more edges become randomly re-connected and finally for p = 1 all connections are random. Thus, this simple model allows to investigate the whole range from regular to random networks, including an intermediate range. The intermediate range proved to be crucial to the solution of the problem.

To show this, the authors introduced two measures: the clustering coefficient C, which is the likelihood that neighbours of a vertex will also be connected, and the path length L which is the average of the shortest distance between pairs of vertices counted in number of edges. Watts and Strogatz showed that regular networks have a high C but also a very high L. In contrast, random networks have a low C and a low L. So, neither regular nor random networks explain the small-world phenomenon. However, when p is only slightly higher than 0 (few edges randomly rewired) the path length L drops sharply, while the clustering coefficient hardly changes. Thus networks with a small fraction of randomly rewired connections combine both high clustering and a small path length, and this is exactly the small-world phenomenon to be explained. These networks were called 'small-world' networks by the authors, who showed that such networks could be found in the nervous system of C. elegans, a social network of actors and the network of power plants in the United States. Also, they showed that a small-world architecture might facilitate the spread of infection or information in networks.

A second major discovery was made a year later by Barabasi and Albert [17]. They proposed a model for the growth of a network where the likelihood that a newly added edge will connect to a vertex depends upon the degree of this vertex. Thus, vertices that have a high degree (large number of edges) are more likely to get even more edges. This is the network equivalent of 'the rich getting richer'. Networks generated in this way are characterised by a degree distribution which can be described by a power law: P(k) = k^-1/a^. In the case of the Barabasi Albert model the exponent is exactly 3. Networks with a power law degree distribution are called scale-free. It has been shown that many real networks in nature such as for instance the Internet, the World Wide Web, collaboration networks of scientists and networks of airports are likely to be scale-free [27,28]. Scale-free networks have many interesting properties such as an extremely short path length, which will be discussed in the section below.

The discovery of small-world networks in 1998 and of scale-free networks in 1999 was noted by scientists in many different fields, and set off a large body of theoretical and experimental research that is growing to this day. In retrospect these discoveries can be considered to be the starting point of the modern theory of networks. The field is so new that there are only few textbooks yet [28,29]. Fortunately there are several excellent reviews that give an overview of the current state of network theory [27,30-33]. A collection of key papers can be found in Newman et al. [34].

## 3. Basics of modern network theory

### 3.1 Definition of graphs and graph measures

**Figure 1:**
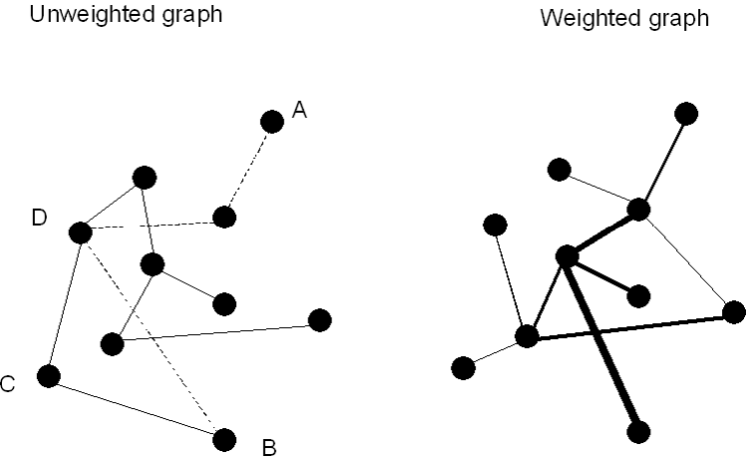
Representation of a network as a graph. In the case of an unweighted graph (left panel) black dots represent the nodes or vertices, and the lines connecting the dots the connections or edges. The shortest path between vertices A and B consists of three edges, indicted by the striped lines. The clustering coefficient of a vertex is the likelihood that its neighbours are connected. For vertex C, with neighbours B and D, the clustering coefficient is 1. When weights are assigned to the edges, the graph is weighted (right panel). Here the weights of the edges are indicated by the thickness of the lines.

A graph is an abstract representation of a network. It consists of a set of vertices (or nodes) and a set of edges (or connections) (Fig. 1). The presence of an edge between two vertices indicates the presence of some kind of interaction or connection between the vertices (the interpretation depends upon what is being modelled with the graph). The adjacency matrix A contains the information about the connectivity structure of the graph. When an edge exists between two vertices i and j the corresponding entry of the adjacency matrix is: A_i,j _= 1; otherwise A_i,j _= 0. The number of edges connecting to ('incident on') a vertex is called the degree k of this vertex. The likelihood P(k) that a randomly chosen vertex will have degree k is given by the degree distribution: it is a plot of P(k) as a function of k. The degree distribution can have different forms: Gaussian, binomial, Poisson, exponential or power law. The degree distribution is an important determinant of network properties.

With respect to the edges several further distinctions can be made. Graphs can be undirected, when information can flow in both directions along edges connecting vertices, or directed, when information can only flow in one direction. In directed graphs each vertex may have different numbers of ingoing and outgoing edges; correspondingly there are separate in degree and out degree distributions for such graphs. Graphs which contain vertices connected by more than one edge are called multigraphs. Graphs in which edges either exist or do not exist, and in which all edges have the same significance are called unweighted graphs. When weights are assigned to each of the edges the corresponding graph is called a weighted graph (right panel in Fig. 1). Weights can be used to indicate the strength or effectiveness of connections, or the distance between vertices; negative weights can also be used.

Two measures are frequently used to characterize the local and global structure of unweighted graphs [16,27,33]. These are the clustering coefficient C and the characteristic path length L. The clustering coefficient C_i _of a vertex i with degree k_i _is usually defined as the ratio of the number of existing edges (e_i_) between neighbours of i, and the maximum possible number of edges between neighbours if i. A vertex is called a neighbour of i when it is connected to it by an edge. The formula for C_i _is:

Ci=2eiki(ki−1)=∑j,mai,jaj,mam,iki(ki−1).
 MathType@MTEF@5@5@+=feaafiart1ev1aaatCvAUfKttLearuWrP9MDH5MBPbIqV92AaeXatLxBI9gBaebbnrfifHhDYfgasaacH8akY=wiFfYdH8Gipec8Eeeu0xXdbba9frFj0=OqFfea0dXdd9vqai=hGuQ8kuc9pgc9s8qqaq=dirpe0xb9q8qiLsFr0=vr0=vr0dc8meaabaqaciaacaGaaeqabaqabeGadaaakeaacqWGdbWqdaWgaaWcbaGaemyAaKgabeaakiabg2da9maalaaabaGaeGOmaiJaemyzau2aaSbaaSqaaiabdMgaPbqabaaakeaacqWGRbWAdaWgaaWcbaGaemyAaKgabeaakiabcIcaOiabdUgaRnaaBaaaleaacqWGPbqAaeqaaOGaeyOeI0IaeGymaeJaeiykaKcaaiabg2da9maalaaabaWaaabuaeaacqWGHbqydaWgaaWcbaGaemyAaKMaeiilaWIaemOAaOgabeaakiabdggaHnaaBaaaleaacqWGQbGAcqGGSaalcqWGTbqBaeqaaOGaemyyae2aaSbaaSqaaiabd2gaTjabcYcaSiabdMgaPbqabaaabaGaemOAaOMaeiilaWIaemyBa0gabeqdcqGHris5aaGcbaGaem4AaS2aaSbaaSqaaiabdMgaPbqabaGccqGGOaakcqWGRbWAdaWgaaWcbaGaemyAaKgabeaakiabgkHiTiabigdaXiabcMcaPaaacqGGUaGlaaa@5E21@

A slightly different definition can be found in Newman (Newman, 2003). The clustering coefficient C ranges between 0 and 1. Usually C_i _is averaged over all vertices to obtain a mean C of the graph.

C=〈c〉=1N∑i=1Nci.
 MathType@MTEF@5@5@+=feaafiart1ev1aaatCvAUfKttLearuWrP9MDH5MBPbIqV92AaeXatLxBI9gBaebbnrfifHhDYfgasaacH8akY=wiFfYdH8Gipec8Eeeu0xXdbba9frFj0=OqFfea0dXdd9vqai=hGuQ8kuc9pgc9s8qqaq=dirpe0xb9q8qiLsFr0=vr0=vr0dc8meaabaqaciaacaGaaeqabaqabeGadaaakeaacqWGdbWqcqGH9aqpdaaadaqaaiabdogaJbGaayzkJiaawQYiaiabg2da9maalaaabaGaeGymaedabaGaemOta4eaamaaqahabaGaem4yam2aaSbaaSqaaiabdMgaPbqabaaabaGaemyAaKMaeyypa0JaeGymaedabaGaemOta4eaniabggHiLdGccqGGUaGlaaa@3F7C@

The clustering coefficient is an index of local structure, and has been interpreted as a measure of resilience to random error (if vertex i is lost, its neighbours remain still connected).

Another important measure is the characteristic path length. In the case of an unweighted graph the path length or distance d_i,j _between two vertices i and j is the minimal number of edges that have to be travelled to go from i to j. This is also called the geodesic path between i and j. The characteristic path length L of a graph is the mean of the path lengths between all possible pairs of vertices:

L=1N(N−1)∑i,j∈N,i≠jdi,j.
 MathType@MTEF@5@5@+=feaafiart1ev1aaatCvAUfKttLearuWrP9MDH5MBPbIqV92AaeXatLxBI9gBaebbnrfifHhDYfgasaacH8akY=wiFfYdH8Gipec8Eeeu0xXdbba9frFj0=OqFfea0dXdd9vqai=hGuQ8kuc9pgc9s8qqaq=dirpe0xb9q8qiLsFr0=vr0=vr0dc8meaabaqaciaacaGaaeqabaqabeGadaaakeaacqWGmbatcqGH9aqpdaWcaaqaaiabigdaXaqaaiabd6eaojabcIcaOiabd6eaojabgkHiTiabigdaXiabcMcaPaaadaaeqbqaaiabdsgaKnaaBaaaleaacqWGPbqAcqGGSaalcqWGQbGAaeqaaaqaaiabdMgaPjabcYcaSiabdQgaQjabgIGiolabd6eaojabcYcaSiabdMgaPjabgcMi5kabdQgaQbqab0GaeyyeIuoakiabc6caUaaa@4967@

The characteristic path length is a global characteristic; it indicates how well integrated a graph is, and how easy it is to transport information or other entities in the network. A measure related to the path length is the diameter of a graph: this is the length (in number of edges) of the longest geodesic in a graph.

**Figure 2:**
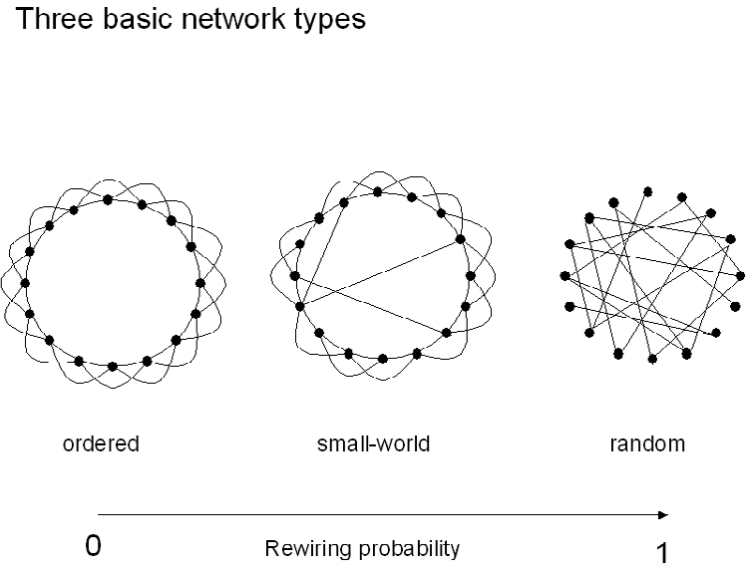
Three basic network types in the model of Watts and Strogatz. The leftmost graph is a ring of 16 vertices (N = 16), where each vertex is connected to four neighbours (k = 4). This is an ordered graph which has a high clustering coefficient C and a long pathlength L. By choosing an edge at random, and reconnecting it to a randomly chosen vertex, graphs with increasingly random structure can be generated for increasing rewiring probability p. In the case of p = 1, the graph becomes completely random, and has a low clustering coefficient and a short pathlength. For small values of p so-called small-world networks arise, which combine the high clustering coefficient of ordered networks with the short pathlength of random networks.

**Figure 3:**
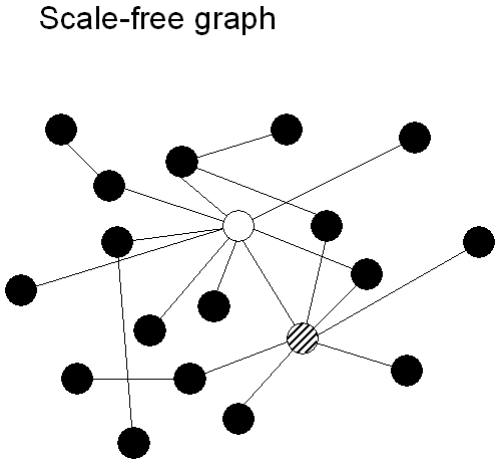
Scale-free graphs are characterized by a scale-free degree distribution P(k). In scale-free graphs, different vertices have very different degrees, and typically a few vertices with extremely high degrees (so-called 'hubs') are present. In the schematic example shown here the white (k = 9) and the striped (k = 7) vertices are examples of hubs.

The degree distribution, clustering coefficient and path length are the core measures of graphs. On the basis of these measures four different types of graphs can be distinguished: (i) ordered or lattice like; (ii) small-world; (iii) random and (iv) scale-free (Fig. 2, 3). A further subdivision is described in Amaral et al. [36]. In an ordered network, each vertex is connected to its k nearest neighbours. What 'nearest' means depends upon the dimension in which the network is modelled. In most cases, one or two dimensional networks are considered. Ordered or lattice like networks have a high C and a large L. For the one-dimensional model of Watts and Strogatz the theoretical values of C and L are 3/4 and N/2K. A small world network can be thought of as an ordered network where a small fraction of the edges (given by the rewiring probability p) has been randomly rewired. Such a network has a C close to that of an ordered network, but a very small path length close to that of a random network. However, analytical solutions of C and L as a function of p are not known [27]. In a random network, all edges are randomly assigned to vertex pairs (or: edges exist with a certain likelihood). In a random network, C is very small (K/N) and L is very short: ln(N)/ln(K). Finally, a scale-free network is a network with a power law degree distribution. Such a network could be generated by a growth process characterized by preferential attachment (Barabasi and Albert, 1999). However, other growth models for scale-free networks have been proposed [27,33]. We should stress that neither lattice like, small-world or random networks are scale-free. Scale-free networks can have very small path lengths of the order of lnln(N), but the clustering coefficient may also be smaller than that of small-world networks [36].

In addition to clustering coefficients, pathlengths and degree distributions other measures have been introduced to characterize properties of interest. Milo et al. introduced the concept of network motifs [37,38]. A motif is a simple subgraph consisting of a small number of vertices connected in a specific way. Triangles are a simple type of motif. To some extent, the clustering coefficient is an index of a specific type of motif, namely the triangle. Alternatively, one could view motif analysis as a kind of generalization of the clustering coefficient. Another measure is the degree correlation. This is an index of whether the degree of a vertex is influenced by the degree of another vertex to which it connects. The average degree k_nn _of the neighbours of a node with degree k is given by:

knn(k)=∑k'k'P(k'|k).
 MathType@MTEF@5@5@+=feaafiart1ev1aaatCvAUfKttLearuWrP9MDH5MBPbIqV92AaeXatLxBI9gBaebbnrfifHhDYfgasaacH8akY=wiFfYdH8Gipec8Eeeu0xXdbba9frFj0=OqFfea0dXdd9vqai=hGuQ8kuc9pgc9s8qqaq=dirpe0xb9q8qiLsFr0=vr0=vr0dc8meaabaqaciaacaGaaeqabaqabeGadaaakeaacqWGRbWAdaWgaaWcbaGaemOBa4MaemOBa4gabeaakiabcIcaOiabdUgaRjabcMcaPiabg2da9maaqafabaGaem4AaS2aaWbaaSqabeaacqGGNaWjaaGccqWGqbaucqGGOaakcqWGRbWAdaahaaWcbeqaaiabcEcaNaaakmaaeeaabaGaem4AaSgacaGLhWoacqGGPaqkaSqaaiabdUgaRnaaCaaameqabaGaei4jaCcaaaWcbeqdcqGHris5aOGaeiOla4caaa@4547@

Graphs with a positive degree correlation are called assortative; in the case of a negative degree correlation a graph is called disassortative. Degree correlations can be quantified by computing the Pearson correlation coefficient of the degrees of pairs of vertices connected by an edge. Interestingly, most social networks tend to be assortative, while most technological and biological networks tend to be disassortative (table 3.1 in [27].). An index of the relative importance of a vertex or edge is the 'betweenness'. This is the number of shortest paths that run through an edge or vertex. The beweenness of a node b_i _is defined as:

bi=∑j,k∈N,j≠knj,k(i)nj,k.
 MathType@MTEF@5@5@+=feaafiart1ev1aaatCvAUfKttLearuWrP9MDH5MBPbIqV92AaeXatLxBI9gBaebbnrfifHhDYfgasaacH8akY=wiFfYdH8Gipec8Eeeu0xXdbba9frFj0=OqFfea0dXdd9vqai=hGuQ8kuc9pgc9s8qqaq=dirpe0xb9q8qiLsFr0=vr0=vr0dc8meaabaqaciaacaGaaeqabaqabeGadaaakeaacqWGIbGydaWgaaWcbaGaemyAaKgabeaakiabg2da9maaqafabaWaaSaaaeaacqWGUbGBdaWgaaWcbaGaemOAaOMaeiilaWIaem4AaSgabeaakiabcIcaOiabdMgaPjabcMcaPaqaaiabd6gaUnaaBaaaleaacqWGQbGAcqGGSaalcqWGRbWAaeqaaaaaaeaacqWGQbGAcqGGSaalcqWGRbWAcqGHiiIZcqWGobGtcqGGSaalcqWGQbGAcqGHGjsUcqWGRbWAaeqaniabggHiLdGccqGGUaGlaaa@4CBF@

This is the ratio of all shortest paths between j and k that run through i (n_j,k_(i)), divided by all shortests paths between j and k (n_j,k_). This measure also reflects the consequences of the loss of a particular edge or vertex. Another recently described measure is the transversal time for random walks on small-world networks [39]. Characterization of overlapping communities in complex networks has recently been described by Palla et al. [40].

Most graph measures have only been defined for the simplest case of unweighted graphs. However in many cases weighted graphs may represent more accurate models of real networks. Several authors have discussed the analysis of weighted graphs [41-47]. To characterize such networks one could convert them to unweighted graphs, for instance by setting all edges with a weight above a certain threshold to 1, and the others to 0. Although this approach works and has been used in EEG and MEG studies, it has several disadvantages: (i) much of the information available in the weights is not used; (ii) when the threshold is too high some vertices may become disconnected from the graph which poses problem with the computation of C and L; (iii) the choice of the threshold remains arbitrary. Latora and Marchiori have proposed a framework to address some of these problems [41,42,48]. They consider weighted networks and define the efficiency of the path between two vertices as the inverse of the shortest distance between the vertices (note that in weighted graphs the shortest path is not necessarily the path with the smallest number of edges). In the case where a path does not exist, the length is considered to be infinite, and the efficiency is zero. The average over all pair wise efficiencies is the Global efficiency E_glob _of the graph:

Eglob=1N(N−1)∑i,j∈N,i≠j1di,j.
 MathType@MTEF@5@5@+=feaafiart1ev1aaatCvAUfKttLearuWrP9MDH5MBPbIqV92AaeXatLxBI9gBaebbnrfifHhDYfgasaacH8akY=wiFfYdH8Gipec8Eeeu0xXdbba9frFj0=OqFfea0dXdd9vqai=hGuQ8kuc9pgc9s8qqaq=dirpe0xb9q8qiLsFr0=vr0=vr0dc8meaabaqaciaacaGaaeqabaqabeGadaaakeaacqWGfbqrdaWgaaWcbaGaem4zaCMaemiBaWMaem4Ba8MaemOyaigabeaakiabg2da9maalaaabaGaeGymaedabaGaemOta4KaeiikaGIaemOta4KaeyOeI0IaeGymaeJaeiykaKcaamaaqafabaWaaSaaaeaacqaIXaqmaeaacqWGKbazdaWgaaWcbaGaemyAaKMaeiilaWIaemOAaOgabeaaaaaabaGaemyAaKMaeiilaWIaemOAaOMaeyicI4SaemOta4KaeiilaWIaemyAaKMaeyiyIKRaemOAaOgabeqdcqGHris5aOGaeiOla4caaa@4FFB@

The Local efficiency is the mean of the efficiencies of all subgraphs G_i _of neighbours of each of the vertices of the graph. The average local efficiency E_loc _is given by:

Eloc=1N∑i∈NE(Gi).
 MathType@MTEF@5@5@+=feaafiart1ev1aaatCvAUfKttLearuWrP9MDH5MBPbIqV92AaeXatLxBI9gBaebbnrfifHhDYfgasaacH8akY=wiFfYdH8Gipec8Eeeu0xXdbba9frFj0=OqFfea0dXdd9vqai=hGuQ8kuc9pgc9s8qqaq=dirpe0xb9q8qiLsFr0=vr0=vr0dc8meaabaqaciaacaGaaeqabaqabeGadaaakeaacqWGfbqrdaWgaaWcbaGaemiBaWMaem4Ba8Maem4yamgabeaakiabg2da9maalaaabaGaeGymaedabaGaemOta4eaamaaqafabaGaemyrauKaeiikaGIaem4raC0aaSbaaSqaaiabdMgaPbqabaGccqGGPaqkaSqaaiabdMgaPjabgIGiolabd6eaobqab0GaeyyeIuoakiabc6caUaaa@41B9@

The approach based upon efficiencies is attractive since it takes into account the full information contained in the graph weights, and provides an elegant solution to handle disconnected vertices. Efficiency has been used to show that scale-free networks are very resistant to random errors, but quite sensitive to targeted attacks [49]. By taking the harmonic mean of the inverse of the efficiencies a weighted path length can be defined, which is a bit closer to the original path length (formula 3.2 in [27].). Slightly modified the formula is:

L−1=1N(N−1)∑i,j∈N,i≠j1di,j.
 MathType@MTEF@5@5@+=feaafiart1ev1aaatCvAUfKttLearuWrP9MDH5MBPbIqV92AaeXatLxBI9gBaebbnrfifHhDYfgasaacH8akY=wiFfYdH8Gipec8Eeeu0xXdbba9frFj0=OqFfea0dXdd9vqai=hGuQ8kuc9pgc9s8qqaq=dirpe0xb9q8qiLsFr0=vr0=vr0dc8meaabaqaciaacaGaaeqabaqabeGadaaakeaacqWGmbatdaahaaWcbeqaaiabgkHiTiabigdaXaaakiabg2da9maalaaabaGaeGymaedabaGaemOta4KaeiikaGIaemOta4KaeyOeI0IaeGymaeJaeiykaKcaamaaqafabaWaaSaaaeaacqaIXaqmaeaacqWGKbazdaWgaaWcbaGaemyAaKMaeiilaWIaemOAaOgabeaaaaaabaGaemyAaKMaeiilaWIaemOAaOMaeyicI4SaemOta4KaeiilaWIaemyAaKMaeyiyIKRaemOAaOgabeqdcqGHris5aOGaeiOla4caaa@4C7B@

Apart from the Local efficiency, two other definitions of the clustering coefficient have been proposed for weighted networks. In one definition only the weights of the edges connecting the neighbours of a vertex are taken into account, while the edges connecting this vertex to its neighbours are all given a weight of 1 [46]. It is also possible to define a weighted clustering coefficient, that takes into account both the weights between the reference vertex and its neighbours, as well as the weights of the edges between the neighbours [47]. In the last study an approach to the analysis of motifs in weighted graphs was also proposed.

Finally we briefly mention a measure of the 'synchronizability' of a graph. This measure is based upon a so-called linear stability analysis. A detailed description can be found in [33]. Briefly, the spectrum of eigenvalues from the graph laplacian L is determined. This matrix L is the difference between the diagonal matrix of node degrees and the adjacency matrix A. The eigenvalues are ordered from the largest to the smallest, where λ_1 _= 0. The ratio R = λ_N_/λ_2 _of the largest and one but smallest eigenvalue is a measure of the synchronizability of the graph. This approach has been used for unweighted as well as weighted networks, and will be referred to in the studies discussed in the following section.

### 3.2 Dynamic processes on graphs

One of the most interesting and active research areas in modern network theory is the study of structure function relationships, in particular the relation between topological network characteristics and synchronization dynamics on these networks [50]. The importance of the small-world structure for the spread of infectious disease was already addressed in the original Watss and Strogatz paper [16]. Barahona and Pecora used linear stability analysis and the master stability function (MSF) to study the synchronizability of networks with complex topology [51]. They showed that networks with a small-world topology may synchronize more easily than deterministic or fully random graphs, although the presence of small-world properties did not guarantee that the network will be synchronizable. Hong et al. studied the synchronization dynamics of a small-world network of coupled oscillators as a function of rewiring probability p [52]. They found that phase and frequency synchronization arise even for small values of p. The phase transition was of the mean field type, like in the Kuramoto model. For values of p > 0.5 the small-world model synchronized as rapidly as a fully random network.

A surprising phenomenon, later referred to as 'the paradox of heterogeneity' was discovered by Nishikawa et al. [53]. Using linear stability analysis and the ratio λ_N_/λ_2 _(largest divided by second smallest eigenvalue of the graph Laplacian matrix) as an index of synchronizability, they showed that (unweighted, undirected) networks with a more homogenous degree distribution synchronize more easily than networks with a more heterogeneous degree distribution, even when the latter network type has a shorter average path length. This observation implied that the previous idea that synchronizability was directly related to path length had to be rejected. The authors explain the paradoxical effect of heterogeneous degree distributions on synchronizability by the 'overload' of the few highly connected nodes in the network.

Another factor with a somewhat unexpected influence on network synchronization is the existence of delays between the coupled dynamic units. Atay and Jost showed in a model of coupled logistic maps that networks with scale-free or random topology could still synchronize despite the delays, whereas lattice like and small-world networks did not synchronize well [54]. However, and this was somewhat unexpected, in some cases where the un delayed network did not synchronize, synchronization did occur when delays were introduced. We should add however that it is not clear to what extent these results obtained with discrete maps can be extrapolated to more general systems of coupled oscillators. In a later paper Atay and Biyikoglu studied systematically the effect of a broad range of graph operations (Cartesion product, join, coalescense, adding/deleting links) on network synchronizability [55]. Especially interesting results were obtained in the case of adding links to networks. First, in some cases adding links between two networks was shown to increase the synchronizability of the individual networks while decreasing the synchronizability of the combined network. Also, adding links to a single network could result in smaller path lengths but at the same time decreased synchronizability. Of course this is reminiscent of the findings of Nishikawa et al. [53]. although the authors claim that the degree distribution of a network in general does not determine its synchronizability. Removing links from networks can also be used to study community structures in networks [43].

Taking the original result of [53]. to the extreme, one would expect that a network with a maximally homogeneous structure would show the highest level of synchronizability. Donetti et al. (2005) described an algorithm to generate such 'hyper homogeneous' networks which they baptized 'entangled networks' [56]. Entangled networks were shown to be optimal not only in terms of synchronizability, but also with respect to resilience against attacks and error. However, the authors state that a full topological understanding of entangled networks has not yet been reached. Synchronizability of scale-free networks of limit cycle oscillators was studied in detail using linear stability analysis by Lee [57]. He found a critical coupling strength for scale-free networks that was smaller than for small-world or random networks. The nature of the synchronization transition depended upon the scaling exponent, and showed a different behaviour for the range 2< exponent<3 as compared to exponent > 3. A relationship between the scaling exponent of the degree distribution and pattern formation in scale-free networks has also been reported by Zou and Lipowsky [58].

An important breakthrough with respect to the 'paradox of heterogeneity' was achieved by Motter et al. [59]. They considered directed, weighted networks, where the weights of the edges were based (with a parameter β) upon the degrees of the nodes. They showed that in the case of weighted, directed networks as opposed to unweighted undirected networks a heterogeneous degree distribution could be associated with an optimal level of synchronizability. The most optimal results, both in terms of synchronizability as well as 'wiring cost' were obtained for β = 1. In contrast for β = 0 (the unweighted case) the results of [53]. were reproduced. The authors also suggested that for large sufficiently random networks the synchronizability is mainly determined by the mean degree, and not by the degree distribution or system size.

Taking this approach one step further, Chavez et al. showed that network synchronizability could be enhanced even more by basing the network weights upon the 'load' (fraction of shortest paths using a particular link: see 'betweenness' b_i _defined in section 3.1) of the links [60,61]. Chavez et al. showed that, in the case of weighted networks, scale-free networks have the highest synchronizability, followed (in order of decreasing synchronizability) by random, small-world and regular/lattice network [60,61]. For small-world networks, synchronizability was shown to increase with the probability of rewiring. Numerical analysis showed that these results obtained with linear stability analysis might hold as well for systems of non-identical oscillators. In particular, the eigenvalue ratio λ_N_/λ_2 _could be a useful indicator of synchronizability even for these networks.

Zhou et al. also studied the synchronizability of weighted random networks with a large minimum degree (k_min _>> 1) [62]. They showed that the synchronizability was mainly determined by the average degree and the heterogeneity of the node's intensity. Intensity is the sum of the strengths of all inputs of a node, and reflects the degree as well as the link weights. Synchronizability was enhanced when the heterogeneity of the nodes intensities was reduced. In a subsequent study Zhou and Kurths investigated whether optimal weights for synchronizability could emerge in adaptive networks [63]. They showed that this was indeed the case in scale-free networks of coupled chaotic oscillators, and that the final weights were negatively correlated with the node's degrees. The adapation process enhanced network synchronizability by reducing the heterogeneity of node intensities. Van den Berg and van Leeuwen also studied the adaptation process and showed that sparsely connected random graphs above a certain size always give rise to a small-world network [64]. In a later study these authors showed that under the influence of an adaptive rewiring procedure a network of randomly connected Hindmarsh-Rose neurons will evolve towards a small-world architecture with complex dynamics [65]. This result was obtained irrespective of the initial dynamics of the network (irregular firing or bursting behaviour).

Synchronization in a complex network of coupled oscillators was studied from a different perspective by Arenas et al. [66]. They showed that a relation exists between the complex, hierarchical structure in the connectivity matrix on the one hand, and different time scales of the synchronization dynamics on the other hand. More specifically, for short time scales the nodes are disconnected, while for longer time scales the nodes become synchronized in groups according to their topological structure. This study underscores once more the importance of structure function relationships in complex networks. Related results were obtained by Zemanova et al. and Zhou et al. [67,68].

## 4. Applications to neuroscience

### 4.1 Dynamics of simulated neural networks

From the previous sections is has become clear that a major research focus in modern network studies is the relation between network topology on the one hand, and dynamics on networks on the other hand. This problem is of major interest for neuroscience, and an important question is to what extent the results obtained with models of general types of oscillators are relevant for networks of neuron-like elements as well.

Lago-Fernandez et al. were the first to study this question in a network of non-identical Hodgkin and Huxley neurons coupled by excitatory synapses [69]. They studied the influence of three basic types of network architecture on coherent oscillations of the network neurons. Random networks displayed a fast system response, but were unable to produce coherent oscillations. Networks with regular topology showed coherent oscillations, but no fast signal processing. Small-world networks showed both a fast system response as well as coherent oscillations, suggesting that this type of architecture could be optimal for information processing in neural networks.

The influence of complex connectivity on neuronal circuit dynamics was also studied by Roxin et al. [70]. They considered a small-world network of excitable, leaky integrate-and-fire neurons. For low values of p (the likelihood of random rewiring) a localized transient stimulus resulted either in self sustained persistent (mostly periodic) activity or a brief transient response. For high values of p, the network displayed exceedingly long transients and disordered patterns. The probability of failure (a stimulus not resulting in sustained activity) showed a phase transition over a small range of values of p. The authors concluded that this 'bi-stability' of the network might represent a mechanisms for short term memory.

Masuda and Aihara showed that in a model of 400 coupled leaky integrate-and-fire neurons small p values gave rise to travelling waves or clustered states, intermediate values to rapid communications between remote neurons and global synchrony, and high p to asynchronous firing [71]. They also showed that network dynamics can be influenced by the degree distribution. With so-called 'balanced rewiring' (same degree for all vertices) the optimal p for synchronization vanished. Increasing p replaced precise local with rough global synchrony.

Synchronization of neurons in networks is important for normal functioning, in particular information processing, but may also reflect abnormal dynamics related to epilepsy. Three modelling studies have addressed this issue specifically. Netoff et al. started from the observation that in a hippocampal slice model of epilepsy the CA3 regions shows short bursts of activity whereas the CA1 regions shows seizure like activity lasting for seconds [72]. To explain these observations they constructed models (small-world networks with N = 3000; k = 30 for CA1 and k = 90 for CA3) of various types of neurons (Poisson spike train, leaky integrate-and-fire, stochastic Hodgkin and Huxley). For increasing values of the rewiring probability, the models displayed first normal behaviour, then seizure like transients and finally continuous bursting. Increasing the strength of the synapses had a similar effect as increasing p. For the CA3 model (with higher k) the transition from seizures to bursting occurred for a lower value of p compared to the CA1 model. These findings suggest that the bursting behaviour of the CA3 region may represent a dynamical state beyond seizures. This is an important suggestion since similar bursting-like phenomena have also been observed in the scalp recorded EEGs of neurological patients, and their epileptic significance is still poorly understood [73].

Percha et al. started with the observation that in medial temporal lobe epilepsy, epileptogenesis is characterized by structural network remodelling and aberrant axonal sprouting [75]. To study the influence of modified network topology on seizure threshold they considered a two-dimensional model of 12 by 12 Hindmarsh-Rose neurons. For increasing values of p they found a phase transition between a state of local to a state of global coherence; the transition occurred at p = 0.3. At the phase transition point the duration of globally coherent states displayed a power law scaling, consistent with type III intermittency. The authors speculated that neural networks may develop towards a critical regime between local and global synchronization; seizures would result if pathology pushes the system beyond this critical state. A similar concept can be found in two other studies [5,75].

The influence of temporal lobe architecture on seizures was also studied by Dyhrfjeld-Johnsen et al. [76]. They studied a computational model of rat dentate gyrus with 1 billion neurons, and no more than three synapses between any two neurons, suggestive of a small-world architecture. They showed that loss of long distance hilar cells had only little influence on global network connectivity as long as a few of these long distance connections were preserved. Also, local axonal sprouting of granular cells resulted in increased local connectivity. Simulations of the dynamics of this model showed that network hyperexcitability was preserved despite the loss of hilar cells.

To explain the dynamics of cultured neural networks French and Gruenstein investigated two-dimensional excitatory small-world networks with bursting integrate-and-fire neurons with regular spiking (RS) and intrinsic bursting (IB) [77]. The model showed spontaneous activity, similar to cultured networks. Traces of membrane potential and cytoplasmatic calcium matched those of experimental data. For even low values of rewiring probability the values for the speed of propagation in the model were within the range of the physiological model. For higher p and more long distance connections wave speed increased. Recently it has been shown that real neural networks cultured in vitro in multi electrode arrays (MEAs) display functional connectivity patterns with small-world characteristics [78].

Higher values of p are known to be associated with shorted path lengths in the Watts and Strogatz small-world model. That pathlength is an important predictor of network performance, as has been shown recently [79]. These authors investigated a two-dimensional lattice of coupled van der Pol-FitzHugh-Nagumo oscillators and considered as measures of network performance: activity and synchronization. They found that network performance was mainly determined by the network average path length: the shorter the path length, the better the performance. Local properties such as the clustering coefficient turned out to be less important.

The studies discussed above considered networks of excitatory elements only. Shin and Kim studied a network of 1000 coupled FitzHugh-Nagumo (FHN) neurons with fixed inhibitory coupling strength and an excitatory coupling strength that changed with firing [80]. Starting from random initial coupling strengths, this network self-organized to both the small-world and the scale-free network regime by synaptic re-organization and by the spike timing dependent synaptic plasticity (STDP). The optimal balance between excitation and inhibition proved to be crucial, as has been observed in other studies [81].

Paula et al. studied small-world and scale-free models of 2048 sparsely coupled (k = 4) McCulloch and Pitts neurons [82]. In the case of regular topology the model showed non-periodic activity, whereas random topology resulted in periodic dynamics, where the duration of the periods depended on the square root of network size. The transition between aperiodic and periodic dynamics as a function of p was suggestive of a phase transition.

Two other studies provide a link with the topic of anatomical connectivity that will be discussed in more detail in the next section. Zhou et al. and Zemanova et al. investigated the correlations between network topology and functional organization of complex brain networks [67,68]. They modelled the cortical network of the cat with 53 areas and 830 connections as a weighted small-world network. Each node or area in the network was modelled as a sub network of excitable FitzHugh-Nagumo neurons (N = 200; k = 12, SWN topology with p = 0.3; 25% inhibitory neurons; 5% of the neurons receive excitatory connections form other areas). The control parameter was the coupling strength g. For weak coupling the model showed non-trivial organization related to the underlying network topology, that is correlation patterns between time series of activity were closely related to the underlying anatomical connectivity. These results are in agreement with those of Arenas et al. described above [66]. In a recent modelling study a close correspondence between functional and anatomical connectivity was confirmed when the functional connectivity was determined for long time scales [83].

So far, only few studies have studied the relevance of network structure for memory processes in simulated neural networks. Two behaviors of such networks are relevant for memory: auto-associative retrieval and self-sustained localized states ('bumps'). Anishchenko and Treves showed that the auto-associative retrieval requires networks with a structure close to random, while the self-sustained localized states were only found in networks with a very ordered structure [84]. Coincidence of both behaviours in a small-world regime could not be demonstrated in networks with realistic as opposed to simple binary neurons.

### 4.2 Neuroanatomical networks

#### 4.2.1 Real networks

Interestingly, the seminal paper of Watts and Strogatz was also the first example of an application of graph theory to a neuroscientific question [16]. Watts and Strogatz studied the anatomical connectivity of the nervous system of C. elegans, which is the sole example of a completely mapped neural network. This neural network could be represented by a graph with N = 282 and k = 14. Neurons were considered to be connected if they shared a synapse or a gap junction. Analysis of this graph yielded a path length L = 2.65 (random network: 2.25) and a clustering coefficient C = 0.28 (random network = 0.05). This represents the first evidence of small-world architecture of a real nervous system.

That similar conclusions can be drawn for nervous systems of vertebrates and primates, was shown by Hilgetag et al. [85]. They studied compilations of corticocortical connection data from macaque (visual, somatosensory, whole cortex) and cat, and analyzed these data with optimal set analysis, non-parametric cluster analysis, and graph theoretical measures. All approaches showed a hierarchical organization with densely locally connected clusters of areas. Generally, path lengths were slightly larger than those of random networks, while clustering coefficients were twice as large as those of random networks: macaque visual: L = 1.69 (random 1.65) C = 0.594 (random 0.321); macaque somatosensory: L = 1.77 (random 1.72) C = 0.569 (random 0.312); macaque whole cortex: L = 2.18 (random 1.95) C = 0.49 (random 0.159); cat whole cortex: L = 1.79 (random 1.67); C = 0.602 (random 0.302). The authors concluded that cortical connectivity possesses attributes of 'small-world' networks.

This raises the question whether the small-world pattern of anatomical connectivity determines the patterns of functional connectivity. Stephan et al. studied data from 19 papers on the spread of (epileptiform) activity after strychnine-induced dysinhibition in macaque cortex in vivo [86]. Graph analysis of functional connectivity networks gave the following results L = 2.1730 (random: 2.1500); C = 0.3830 (random: 0.0149). This represents the first proof of a small-world pattern in functional connectivity data, and suggests a relation between anatomical and functional connectivity patterns. While the study of Stephan et al. was based upon data from the literature, Kotter and Sommer modelled the propagation of epileptiform activity in a large scale model of the cortex of the cat and compared the results with randomly connected networks [87]. They concluded that association fibres and their connections strengths were useful predictors of global topographic activation patterns in the cerebral cortex and that a global structure – function relationship could be demonstrated.

Sporns and Zwi studied data sets of macaque visual and whole cortex, and cat cortex, comparing the results to both lattice and random networks, where the in and out degrees of all vertices were preserved [88]. They computed scaled values of L and C (that is: L and C related to L and C of random networks) and looked for cycles. For all three networks the scaled C was close to that of a lattice network, while the scaled L was close to random networks. They also found that there was little or no evidence for scale-free degree distributions, which makes sense in view of the relatively constant number of 10^4 ^synapses per neuron. According to the authors the small-world architecture of the cortex must play a crucial role in cortical information processing.

Some of the same data studied in the above mentioned papers were re-investigated for the presence of motifs (connected graphs forming a subgraph of a larger network) by Sporns and Kotter [89]. The authors distinguished between structural motifs of size M (specific set of M vertices linked by edges) and functional motifs (same M vertices, but not all edges). Graphs were compared to lattice and random networks which preserved the in and out degree of all vertices. The authors concluded that brain networks maximize both the number and diversity of functional motifs, while the repertoire of structural motifs remains small.

Kaiser and Hilgetag studied the edge vulnerability of macaque and cat cortex, protein- protein interaction networks, and transport networks [90]. Comparisons were made with random and scale-free networks. The average shortest path length was used as a measure of network integrity, and four different measures were used to identify critical connections in the network. Of these, the edge frequency (the fraction of shortest paths using a specific edge; related to the 'betweenness' discussed in section 3.1) was the best measure to predict the influence of deleting an edge on average path length. However, for random and scale-free networks all measures performed not very well. Assuming that biological networks are more likely to be small-world, the edge frequency underscores the importance (for overall network performance) and vulnerability of inter-cluster connections. This conclusion is an agreement with Buzsaki et al. who stressed the importance of long-range interneurons for network architecture and performance [91]. Similarly, Manev and Manev suggested that neurogenesis might give rise to new random connections subserving the small-world properties of brain networks [92].

Extending the work of Watts and Strogatz and Hilgetag et al., Humphries et al. investigated whether a specific sub-network of the brain, the brainstem reticular formation, displays a small-world like architecture [93]. They considered two models based upon neuro-anatomical data: a stochastic and a pruning model, and used a small-world metric defined as: S = (C/C-r)/(L/L-r). Here, C-r and L-r refers to the clustering coefficient and path length of corresponding ensembles of random networks. They found that both models fulfil criteria for a small-world network (high S) for a range of parameter settings; however, the in degree and out degree distributions did not follow a power law, arguing against a scale-free architecture.

The first more or less direct proof of small-world like anatomical connectivity in human was reporter by He et al. [94]. They studied MRI scans of 124 healthy subjects, and assumed that two regions were connected if they displayed statistically significant correlations in cortical thickness. For this analysis the entire cortex was segmented into 54 regions. With this approach, the authors could show that the human brain networks has the characteristics of a small-world network with γ (C/C-r) = 2.36 and λ (L/L-r)= 1.15 and a small-world parameter σ (same as S defined above) = 2.04. Furthermore, the degree distribution corresponded to an exponentially truncated power law, as described by Achard et al. [95].

#### 4.2.2 Theoretical and modelling approaches

Supplementing the empirical studies on neuro-anatomical connectivity several studies have studied the significance of connectivity patterns in complex networks from a more theoretical and modelling based perspective [96]. In particular, Sporns and colleagues have inspired a new approach called 'theoretical neuro-anatomy' [97]. They have pointed out that brains are faced with two opposite requirements: (i) segregation, or local specialization for specific tasks; (ii) integration, combining all the information at a global level [98]. One of the key questions is which kind of anatomical and functional architecture allows segregation and integration to be combined in an optimal way. Sporns et al. studied network models that were allowed to develop to maximize certain properties. Networks which developed when optimising for complexity (defined as an optimal balance between segregation and integration: see [99].) showed small-world characteristics; also the graph theoretical measures of these networks were similar to those of real cortical networks, as described under 4.2.1. [98]. Furthermore, networks selected for optimal complexity had relatively low 'wiring costs'. The authors speculate that this type of network architecture (complex or small-world like) could emerge as an adaptation to rich environments [97,99]. In a later review the authors argued that the emergent complex, small-world architecture of cortical networks might promote high levels of information integration and the formation of a so-called 'dynamic core' [21]. This 'dynamic core' could be a potential substrate of higher cognition and consciousness.

The notion of an optimal architecture has also been studied in terms of wiring costs and optimal component placement in neural networks. Karbowski hypothesized that cerebral cortex architecture is designed to save available resources [100]. In a model he studied the trade off between minimizing energetic and biochemical costs (axonal length and number of synapses). The model showed some similarity with small-world networks, but in contrast to these had a distance-dependent probability of connectivity. Kaiser and Hilgetag studied the well known anatomical networks of macaque cortex, and C. Elegans [101]. They showed that optimal component placement could substantially reduce wiring length in all tested networks. However, in the minimally rewired networks the number of processing steps along the shortest paths would increase compared to the real networks. They concluded that neural networks are more similar to network layouts that minimize length of processing paths rather than wiring length. A different conclusion was reached by Chen et al. who studied wiring optimisation of 278 non-pharyngeal neurons of C. Elegans [102]. They solved for an optimal layout of the network in terms of wiring costs and found that most neurons ended up close to their actual position. However, these authors also noted that some neurons got a new position which strongly deviated from the original one, suggesting the involvement of other biological factors. One might speculate that at least one of the other factors could be an optimal architecture in terms of processing steps as suggested by Kaiser and Hilgetag [101].

### 4.3 Functional networks

**Figure 4:**
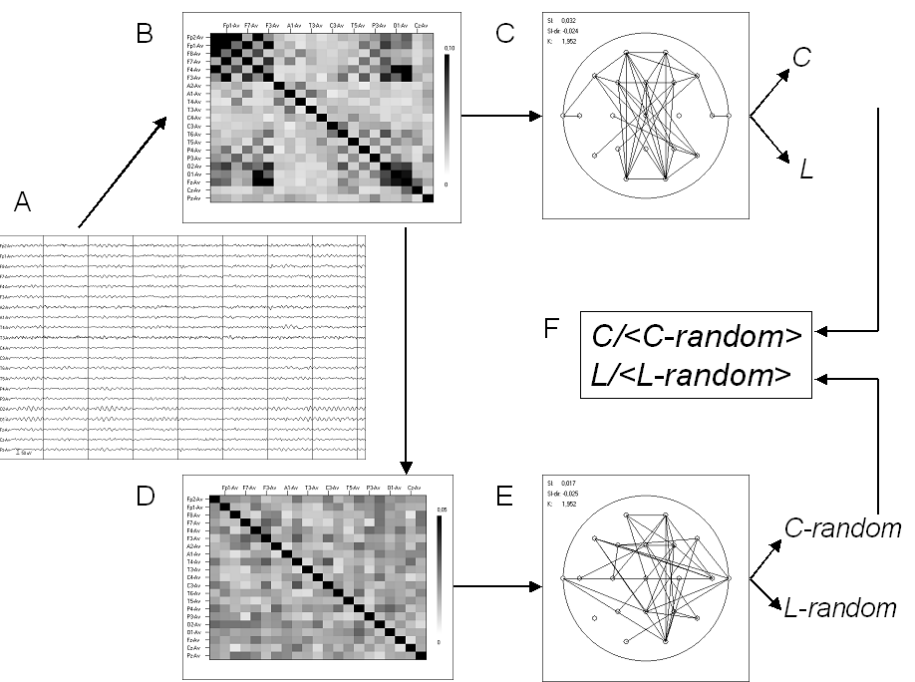
Schematic illustration of graph analysis applied to multi channel recordings of brain activity (fMRI, EEG or MEG). The first step (panel A) consists of computing a measure of correlation between all possible pairs of channels of recorded brain activity. The correlations can be represented in a correlation diagram (panel B, strength of correlation indicated with black white scale). Next a threshold is applied, and all correlations above the threshold are considered to be edges connecting vertices (channels). Thus, the correlation matrix is converted to a unweighted graph (panel C). From this graph various measures such as the clustering coefficient *C *and the path length L can be computed. For comparisons, random networks can be generated by shuffling the cells of the original correlation matrix of panel B. This shuffling preserves the symmetry of the matrix, and the mean strength of the correlations (panel D). From the random matrices graphs are constructed, and graph measures are computed as before. The mean values of the graph measures for the ensemble of random networks are determined. Finally, The ratio of the graph measures of the original network and the mean values of the graph measures of the random networks can be determined (panel F).

The following section on fMRI, EEG, and MEG discusses applications of graph theory to recordings of brain physiology rather than brain anatomy. This approach is based upon the concept of functional or effectivy connectivity, first introduced by Aertsen et al. [103]. The basic assumption is that statistical interdependencies between time series of neuronal activity or related metabolic measures reflect functional interactions between neurons and brain regions. Obviously, patterns of functional connectivity will be restricted by the underlying anatomical connectivity, but they do not have to be identical, and may reveal information beyond the anatomical structure. This is illustrated by the fact that functional connectivity patterns can display rapid task-related changes, as illustrated in several studies discussed below. The basic principles of applying graph analysis to recordings of brain activity are illustrated in Fig. 4.

#### 4.3.1 Functional MRI

Probably the first attempt to apply graph theoretical concepts to fMRI was a methodological paper by Dodel et al. [104]. In this methodological study, graph theory was used as a new approach to identifying functional clusters of activated brain areas during a task. Starting from BOLD (blood oxygen level dependent) time series of brain activity, a matrix of correlations between the time series was computed, and this matrix was converted to a (undirected, unweighted) graph by assigning edges to all supra-threshold correlations. With this approach the authors were able to demonstrate various functional clusters in the form of subgraphs during a finger tapping task. The authors noted the problem that the threshold had a significant influence on the results, and that criteria for choosing an optimal threshold should be considered.

Eguiluz et al. were the first to study clustering coefficients, path lengths, and degree distributions in relation to fMRI data [105,106]. They studied fMRI in 7 subjects during three different finger tapping tasks, and derived matrices of correlations coefficients from the BOLD time series. These matrices were thresholded to obtain unweighted graphs. In this study BOLD time series of each of the fMRI voxels were used. The degree distribution was found to be scale-free, irrespective of the type of task considered. Also, the clustering coefficient was four times larger than that of a random network, and the path length was considered 'close to' that of a random network (in fact depending on the threshold it was 2–3 times larger). The authors concluded that the functional brain networks displayed both scale-free as well as small-world features. Since these properties did not depend upon the task, they assumed that graph analysis mainly reveals invariant properties of the underlying networks, which might be in a 'critical' state [106].

A different approach was taken by the Cambridge group who studied fMRI BOLD time series during a 'resting state' with eyes-closed and no task [95,107-109]. In the first study, fMRI was studied in 12 healthy subjects, and BOLD time series were taken from 90 regions of interest (ROI; 45 from each hemisphere); each of these ROIs corresponded to a specific anatomical region [107]. From these 90 time series a matrix of partial correlations was obtained. The threshold was based upon the significance of the correlations, controlling for false positive findings due to the large number of correlations with the false discovery rate (FDR). The authors found a number of strong and significant correlations, both locally as well as between distant (intra- and inter-hemispherical) brain regions. Hierarchical clustering revealed six major systems consisting of four major cortical lobes, the medial temporal lobe, and a subcortical system. In one patient with a lowered consciousness following an ischemic brain stem lesion a reduction of left intrahemispherical and interhemispherical connections was found.

Graph analysis was applied to unweighted graphs using a significance level of p < 0.05 as a threshold for the partial correlation matrix. The clustering coefficient of this graph was 0.25 (random network: 0.12) and the path length 2.82 (random network: 2.58). The ratio C/C-r was 2.08 and the ratio L/L-r was 1.09, both suggestive of a small-world architecture of the resting state functional network. The authors noted that the anatomy did not always predict precisely the functional relationships, and that the resting state connectivity could be a potentially useful marker of brain disease or damage, as illustrated by the patient example. In another study in five subjects the interdependencies between the BOLD time series were studied in the frequency rather than the time domain [108]. Estimators of partial coherence and a normalized mutual information measure were used to construct the graphs. The authors found stronger fronto-parietal connectivity at lower frequencies and involvement of higher frequencies in the case of local connections.

Subsequently an extensive graph analysis of this data set was performed [95]. Here, wavelet analysis was used to study connectivity patterns as a function of frequency band. The corresponding correlation matrices were thresholded at p < 0.05 using FDR. The resulting graphs displayed a single giant cluster of highly connected brain regions (79 out of 90). In this graph the strongest hubs corresponded to the most recently developed parts of heteromodal association cortex. The most clear-cut small-world pattern was found for BOLD data in the frequency range of 0.03–0.06 Hz. The clustering coefficient was 0.53, and the path length was 2.49. The authors also considered a small-world index as proposed by Humphries: (C/C-r)/(L-L-r). This index is expected to be > 1 in the case of a small world network (relatively high C and low L compared to corresponding random networks). In the case of the experimental graph the index was 2.08, consistent with a small-world network. The authors also investigated the resilience of the network to either 'random attack' (removal of randomly chosen vertex) or targeted attack' (removal of largest hubs). They found that the real brain networks were as resistant to random attacks as either random or scale-free networks. In contrast, the real brain networks were more resistant to targeted attacks than scale-free networks. This finding, as well as the absence of power law scaling and arguments from brain development (where hubs develop late rather than early) suggest to the authors that brain networks are not scale-free as had been suggested by Eguiluz et al [105]. The authors conclude that the functional networks revealed by graph analysis of resting state fMRI might represent a 'physiological substrate for segregated and distributed information processing'.

Finally, the global and local efficiency measures as introduced by Latora and Marchiori were applied in an fMRI study in 15 healthy young and 11 healthy old subjects [109]. The subjects were studied during a resting state no-task paradigm, either with placebo treatment or with sulpiride (an antagonist of the dopamine D2 receptor in the brain). The analysis was based upon wavelet correlation analysis of low frequency correlations between BOLD time series of 90 regions of interest followed by thresholding. The efficiency measures were related to a 'cost' factor, defined as the actual number of edges divided by the maximum number of edges possible in the graph. Local and global efficiency, normalized for cost, were shown to be decreased both in the old compared to the young group and in the sulpiride condition compared to the placebo condition. The effect of age on efficiency was stronger and involved more brain regions than the sulpiride effect. These results were similar irrespective whether the analysis was done on unweighted or weighted graphs reconstructed from the correlation matrix.

#### 4.3.2 EEG and MEG

Data derived from functional MRI experiments – whether task related or resting state – are very suitable for graph analysis because of their high spatial resolution, In contrast, spatial resolution is more problematic with neurophysiological techniques such as EEG and MEG. However, these techniques do measure directly the electromagnetic field related to neuronal activity, and have a much higher temporal resolution.

The first application of graph analysis to MEG was published in 2004 [110]. In this experiment MEG recordings of five subjects during a no-task, eyes-closed state were analysed. Correlations between the time series of the 126 artefact-free channels studied were analysed with the synchronization likelihood (SL), a non-linear measure of statistical interdependencies [111,112]. The matrices of pair wise SL values were converted to unweighted graphs by assuming an edge between pairs of channels (vertices) with an SL above a threshold, and no edge in the case of a subthreshold SL. In all cases the threshold was chosen such that the mean degree was 15. This analysis was performed for MEG data filtered in different frequency bands. For intermediate frequencies the corresponding graphs were close to ordered networks (high clustering coefficient, and long path length). For low (< 8 Hz) and high (> 30 Hz) frequencies the graphs showed small-world features with high C and low L. These results were fairly consistent when the degree k was varied between 10 and 20, although both C and L increased as a function of K.

Graph theoretical properties of MEG recordings in healthy subjects were studied more extensively in a recent paper by Bassett et al. [1,113]. The authors applied graph analysis to MEG recordings in 22 healthy subjects during a no-task, eyes-open state and a simple motor task (finger tapping). Wavelet analysis was used to obtain correlation matrices in the major frequency bands ranging from delta to gamma. After thresholding unweighted, undirected graphs were obtained and characterized in terms of an impressive range of graph theoretical measures such as clustering coefficient, path length, small world metric σ ([C/C-random]./[L/L-random]. see [93].), clustering, characteristic length scale, betweenness and synchronizability (although it is not very well described in the paper the authors probably refer to the eigenvalue ratio based upon graph spectral analysis: λ_N_/λ_2_). In all six frequency bands a small world architecture was found, characterized by values of the small world metric σ between 1.7 and 2. This small-world pattern was remarkably stable over different frequency bands as well as experimental conditions. During the motor task relatively small changes in network topology were observed, mainly consisting of the emergence of long distance interactions between frontal and parietal areas in the beta and gamma bands. Analysis of the synchronizability showed that the networks were in a critical dynamical state close to transition between the non-synchronized and synchronized state.

The first application of graph analysis to EEG was published in 2007 [114]. Here a group of 15 Alzheimer patients was compared to a non-demented control group of 13 subjects. EEG recorded from 21 channels during an eyes-closed, no-task state and filtered in the beta band (13–30 Hz) was analysed with the synchronization likelihood. When C and L were computed as a function of threshold (same threshold for controls and patients), the path length was significantly longer in the AD group. For very high thresholds it was noted that the graphs became disconnected, and the pathlength became shorted in the AD group. When C and L were studied as a function of degree k (same K for both groups), the path length was shorter in the AD group, but only for a small range of K (around 3). For both controls and patients the graphs showed small-world features when C and L were compared to those of random control networks (with preserved degree distribution). A higher mini mental state examination score (MMSE) correlated with a higher C and smaller L. The results were interpreted in terms of a less optimal, that is less small-world like network organization in the AD group.

Bartolomei et al. applied graph analysis to MEG resting state recordings in a group of 17 patients with brain tumours and 15 healthy controls. [115]. Unweighted graphs were obtained from SL matrices of MEG filtered in different frequency bands, using an average degree k of 10, and a network size (number of channels) of 149. Mean SL values were higher in patients in the lower frequency bands (delta, theta and alpha), and lower in the higher frequency bands (beta and gamma). In patients the ratio of the clustering coefficient and the mean clustering coefficient for random networks (C/C-r) was lower than in controls in the theta and gamma band (for right sided tumours); the ratio of pathlength and mean pathlength of random networks (L/L-r) was lower in patients in the theta band, the beta band (for left sided tumours) and the gamma band (for right sided tumours). The general pattern that emerges from this study is that pathological networks are closer to random networks, and healthy networks are closer to small-world networks. Interestingly, such random networks might have a lower threshold for seizures (which occur frequently in patients with low grade brain tumours) than small-world networks.

In two related studies Micheloyannis et al. applied graph analysis to 28 channel EEG recorded during an 2-back working memory test [116,117]. In both studies EEG filtered in different frequency bands was analysed with the SL, and converted to unweighted graphs either as a function of threshold, or as a function of degree K (with K = 5). Also, the ratios C/C-r and L/L-r were computed, relating the C and L to those of random networks with the same degree distribution. In the first study 20 healthy subjects with a few years of formal education and a low IQ were compared to 20 healthy subjects with university degrees and a high IQ [116]. Mean SL did not differ between the two groups. Graph analysis of the no-task condition did not show differences between the groups either. However, during the working memory task the networks in the group with lower education as compared to the highly educated group were closer to small-world networks as revealed by a higher C/C-r and a lower L/L-s in the theta, alpha1, alpha2, beta and gamma band. The results were explained in terms of the neural efficiency hypothesis: the lower educated subjects would 'need' the more optimal small-world configuration during the working memory task to compensate for their lower cognitive abilities.

In the second study the 20 control subjects with higher education were compared to 20 patients with schizophrenia (stable disease, under drug treatment). During the working memory task the C/C-r was lower in the schizophrenia group compared to controls in alpha1, alpha2, beta and gamma bands. Consequently, task related networks in the schizophrenia group were less small-world like, and more random compared to controls. Combining these results with those of the first study there is a decrease of small-world features going from controls with low education to controls with high education, and then from controls with high education to schizophrenia patients. One might speculate that the controls with low education display a compensation mechanism during the task, which is not needed by the highly educated controls and which completely fails in the case of the patients. Of interest, the notion of a more random network in schizophrenia has recently been confirmed in a study in 40 patients and 40 controls [118]. In this EEG based study the patients were characterized by a lower clustering coefficient, a shorter path length and a lower centrality index of the major network hubs. It should be noted that the patients in the Micheloyannis et al and the Breakspear et al studies were treated with antipsychotic drugs, and that an influence of the drug treatment on the network features was found in the Breakspear et al study. Thus, the 'network randomization' could reflect both disease as well as pharmacological effects.

The two studies by Micheloyannis et al. [116,117]. and the study by Bassett et al. [113]. showed the influence of a cognitive or motor task on network topology. This raises the question to what extent network features such as C and L reflect 'state' or 'trait' characteristics. In this context, changes during sleep are of interest. Ferri et al. showed that network properties change during sleep [119]. In 10 healthy subjects 19 channel EEG recordings filtered between 0.25–2.5 Hz were analysed with the synchronization likelihood. Unweighted networks were derived from the SL matrices with a fixed K = 3. The ratio C/C-r but not the ratio L/L-r was found to increase during all sleep stages compared to the awake state; however there were no differences between the various sleep stages. When the sleep architecture was studied in more detail taking into account to CAP (cyclic alternating pattern) phases a higher increase in C/C-r during the CAP A1 phase than during CAP B phase was found. Thus networks features can change during a cognitive task as well as under the influence of sleep. However, there is preliminary evidence that network properties have strong 'trait' characteristics as well. Dirk Smit et al. applied graph analysis to no-task EEG recordings in a large sample of 732 healthy subjects, consisting of mono and dizygotic twins and their siblings (Smit et al, 2006) [120]. In a previous study it was already shown that the mean SL has a strong genetic component, especially in the alpha band (Posthuma et al., 2005) [121]. In the study of Smit et al, both C and L in showed substantial and significant heritability in theta, alpha1, alpha2, beta1, beta2 and beta 3 bands. Furthermore, small-world like properties of the theta and beta band connectivity were related to individual differences in verbal comprehension [120].

The change in network properties during a physiological change in level of consciousness such as sleep raises the question whether network properties might also be affected by pathological changes in consciousness such as occur during epileptic seizures. Two modelling studies have pointed at the importance of network topology for spread of epileptic activity in a network [72,74]. A first preliminary report on network analysis of EEG depth recordings in a single patient during an epileptic seizure was published by Wu and Guan [122]. The authors constructed graphs with N = 30 by using both channels (six) and different frequency bands (five) to construct un weighted networks with degrees varying from 4–7. The bispectrum was used to extract phase coupling information form the EEG. During the seizure a change in network configuration was detected in the direction of a small-world network: there was an increase in C and a decrease of L. Conversely, one might argue that the preceding interictal network was relatively more random.

In a larger study Ponten et al. investigated seven patients during temporal lobe seizures recorded with intracranial depth electrodes [123]. EEG time series filtered in various frequency bands were analysed with the synchronization likelihood, and the SL matrices were converted to unweighted graphs with a fixed degree of 6. A slightly modified definition of L was used (L was defined as the harmonic mean instead of the arithmetic mean of the shortest path lengths: see section 3.1 of this paper) which dealt conveniently with the problem of disconnected points. During seizures the ratio C/C-r increased in delta, theta and alpha bands; L/L-r also increased in the same bands. Thus ictal changes reflected a movement away from a random interictal towards a more ordered ictal network configuration. This suggests that epilepsy might perhaps be characterized be interictal networks with a pathological random structure. Such a random structure has an even lower threshold for the spreading of seizures than the normal small-world configuration (random networks are more synchronizable than small-world networks: see [60,61].); the results of Bartolomei et al. [115]. seem to be in agreement with this hypothesis and suggest that 'network randomisation' might be a general result of brain damage. Needless to say that this bold hypothesis has to be explored in further studies.

## 5. Conclusions and future prospects

To conclude this review we would like to draw some conclusions and suggest a number of problems to be addressed by future research. A first important conclusion is that the modern theory of networks, which originated with the discovery of small-world and scale-free networks, is a very useful framework for the study of large scale networks in the brain. There are several reasons for this: (i) the new theory provides us with powerful realistic models of complex networks in the brain; (ii) a large and still increasing number of measures becomes available to study topological and dynamical properties of these networks; (iii) the theory allows us to better understand the correlations between network structure and the processes taking place on these networks, in particular synchronization processes; (iv) by relating structure to function the notion of an optimal network (in terms of balancing segregation and integration, and performance and cost) can be formulated; (v) the theory provides scenario's how complex networks might develop, and how they might responds to different types of damage (random error versus targeted attack). These considerations explain the motivation to apply modern network theory to neuroscience.

A second conclusion is that modelling studies with neural networks underscore importance of structure function relationships suggested by more fundamental work, and point in the direction of systems with a critical dynamics close to onset of synchronization. Of considerable clinical interest is the work suggesting a relationship between network structure and pathological synchronization, providing a possible mechanism for [72,74,122,123].

Thirdly, anatomical studies suggest that neural networks, ranging from the central nervous system of C. elegans to cortical networks in the cat and macaque, may be organized as small-world networks, and that patterns of functional connectivity may follow the same pattern [83,85-87].

Fourth, some preliminary conclusions can be drawn from studies of functional connectivity in humans: (i) most studies point in the direction of a small-world pattern for functional connectivity, although scale-free networks have also been described (Eguiluz et al., 2005); (ii)

the small-world topology of functional brain networks is very constant across techniques, conditions and frequency bands; tasks induce only minor modifications; (iii) the architecture of functional brain networks may reflect genetic factors and is related to cognitive performance; (iv) different types of brain disease can disrupt the optimal small-world pattern, sometimes giving rise to more random networks which may be associated with cognitive problems as well as a lower threshold for seizures (pathological hypersynchronization).

Some of these conclusions may provide useful starting points for future studies. However, any future work in this field will also have to consider a number of methodological issues. For one thing it is not yet clear what is the optimal way to convert functional imaging data (derived from fMRI, EEG or MEG) to graphs for further analysis. In the case of EEG and MEG the influence of volume conduction on graph measures has not been considered, although it is possible that assessment of the clustering coefficient is biased by this. This raises the question whether the analysis should be done in signal or in source space, and if source reconstruction is needed, what algorithm should be used. Another problem is somewhat arbitrary threshold that is needed to convert a matrix of correlations to an unweighted graph. The choice of the threshold remains a bit arbitrary, and studying a whole range of thresholds may raise statistical problems (type II errors) because of the large number of tests that have to be done. One way out may be to model correlation matrices as weighted graphs, taking into account the full information available. However, at this time only few measures are available for weighted graphs. A further problem that frequently occurs when converting matrices of correlations to graphs is the fact that some of the nodes may become disconnected from the network; this presents difficulties in the calculation of clustering coefficients and path lengths. Use of global and local 'efficiency' measures, and harmonic instead of arithmetic means might provide a solution here [27]. A final remark is that the whole spectrum of graph theoretical measures has not yet been explored in most neuroscience studies. An example of study that makes use of a broad range of graph measures is the recent paper by Bassett el al. [113]. Future studies could gain by a careful consideration of all the graph measures which are currently available, and the new measures that are described in physics papers.

Finally, a number of conceptual issues for future studies deserve mentioning. Some of the questions that have to be addressed by new studies are the following: (i) how does network structure change during growth and development? Some theoretical studies have suggested scenario's explaining how small-world or scale-free networks could emerge by activity dependent changes, but whether these scenario's are a proper description of human brain development is an open question; (ii) related to this problem: it is important to know how do genetic and environmental factors influence network features? An influence of genetic factors on network properties in young adults has been suggested, but the underlying mechanisms are completely unknown. (iii) which network factors provide the best explanation for cognitive functioning? It is clear that certain network properties may be associated with increased synchronizability, and that cognition depends upon the formation and dissolution of synchronized networks in the brain? It is not yet known which network properties are the best predictors of cognitive functioning; (iv) is it possible to detect different characteristic scenarios by which brain pathology changes network structure and function? In particular, could it be that different types of brain disease may be related to either 'random error' or 'targeted attack' of brain networks, and is it possible to predict when and how brain disease will give rise to clinical symptoms? Related to this: could a better understanding of neurological disease at the network level give rise to new treatment approaches? (v) is there a relationship between network properties and susceptibility for seizures? Here the hypothesis that brain disease will convert a healthy small-world network to a more random network with a stronger synchronizability, and thus a lower threshold for pathological synchronization/seizures needs further exploration. (vi) is there a relationship between the 'giant cluster' which emerges at the onset of synchronization and consciousness? The relationship between a single cluster of synchronized neurons and brain areas and consciousness has been suggested by several authors [124,125]. Graph theory could extend these ideas by providing an explanation how and when such a giant cluster will appear in neuronal networks, and what properties it is likely to have.
